# Crosstalk between human immunodeficiency virus infection and salivary bacterial function in men who have sex with men

**DOI:** 10.3389/fcimb.2024.1341545

**Published:** 2024-05-08

**Authors:** Ying Guo, Wenjing Wang, Yixi Yu, Xintong Sun, Baojin Zhang, Yan Wang, Jie Cao, Shuo Wen, Xin Wang, Yuchen Li, Siyu Cai, Ruojun Wu, Wenshan Duan, Wei Xia, Feili Wei, Junyi Duan, Haozhi Dong, Shan Guo, Fengqiu Zhang, Zheng Sun, Xiaojie Huang

**Affiliations:** ^1^ Department of Stomatology, Beijing Youan Hospital, Capital Medical University, Beijing, China; ^2^ Clinical and Research Center for Infectious Diseases, Beijing Youan Hospital, Capital Medical University, Beijing, China; ^3^ Center for Clinical Epidemiology and Evidence-Based Medicine, Beijing Children’s Hospital, Capital Medical University, Beijing, China; ^4^ Harvard School of Dental Medicine, Boston, MA, United States; ^5^ Beijing Institute of Hepatology, Beijing Youan Hospital, Capital Medical University, Beijing, China; ^6^ Department of Stomatology, Beijing Daxing District Hospital of Integrated Chinese and Western Medicine, Beijing, China; ^7^ Department of Periodontology, Beijing Stomatological Hospital, Capital Medical University, Beijing, China; ^8^ Department of Oral Medicine, Beijing Stomatological Hospital, Capital Medical University, Beijing, China

**Keywords:** saliva, bacteria, men who have sex with men, HIV, metagenomic analysis

## Abstract

**Background:**

Engaging in anal sexual intercourse markedly increases the risk of developing HIV among men who have sex with men (MSM); oral sexual activities tend to uniquely introduce gut-derived microbes to salivary microbiota, which, combined with an individual’s positive HIV status, may greatly perturb oral microecology. However, till date, only a few published studies have addressed this aspect.

**Methods:**

Based on 16S rRNA sequencing data of bacterial taxa, MicroPITA picks representative samples for metagenomic analysis, effectively revealing how the development and progression of the HIV disease influences oral microbiota in MSM. Therefore, we collected samples from 11 HIV-negative and 44 HIV-positive MSM subjects (stage 0 was defined by HIV RNA positivity, but negative or indeterminate antibody status; stages 1, 2, and 3 were defined by CD4^+^ T lymphocyte counts ≥ 500, 200–499, and ≤ 200 or opportunistic infection) and selected 25 representative saliva samples (5 cases/stage) using MicroPITA. Metagenomic sequencing analysis were performed to explore whether positive HIV status changes salivary bacterial KEGG function and metabolic pathway in MSM.

**Results:**

The core functions of oral microbiota were maintained across each of the five groups, including metabolism, genetic and environmental information processing. All HIV-positive groups displayed KEGG functions of abnormal proliferation, most prominently at stage 0, and others related to metabolism. Clustering relationship analysis tentatively identified functional relationships between groups, with bacterial function being more similar between stage 0-control groups and stage 1-2 groups, whereas the stage 3 group exhibited large functional changes. Although we identified most metabolic pathways as being common to all five groups, several unique pathways formed clusters for certain groups; the stage 0 group had several, while the stage 2 and 3 groups had few, such clusters. The abundance of K03046 was positively correlated with CD4 counts.

**Conclusion:**

As HIV progresses, salivary bacterial function and metabolic pathways in MSM progressively changes, which may be related to HIV promoting abnormal energy metabolism and exacerbate pathogen virulence. Further, infection and drug resistance of acute stage and immune cell destruction of AIDS stage were abnormally increased, predicting an increased risk for MSM individuals to develop systemic and oral diseases.

## 1 Introduction

Owing to exposures during anal intercourse, the prevalence of human immunodeficiency virus (HIV) among men who have sex with men (MSM) is significant (Assi et al., 2019; Tran and Welles, 2019). Indeed, a recent study on MSM in China showed that their odds of HIV infection have trended upwards over time (Dong et al., 2019). HIV can damage gut-associated lymphoid tissue by causing breakdowns of mucosal barriers, which results in both bacterial translocation and dysbiosis. This contributes to a state of persistent inflammation and exacerbated disease progression (Estes et al., 2010; Lozupone et al., 2013; Vázquez-Castellanos et al., 2018; Vujkovic-Cvijin and Somsouk, 2019; Bai et al., 2021). In the case of MSM who also happen to be HIV-positive, anal sex, which can easily disrupt delicate rectal mucosal barriers, is not only a principal viral communicative mechanism among MSM, but also causes significant variation in gut microbiota composition (Noguera-Julian et al., 2016; Li et al., 2019; Tuddenham et al., 2020). Indeed, MSM-derived microbiota tend to be more diverse in composition than non-MSM-derived microbiotas (Noguera-Julian et al., 2016). To date, MSM-derived gut microbiota have no commonly accepted biomarker that is sufficiently robust to accurately predict those MSM with HIV-positive statuses (Nowak et al., 2017; Li et al., 2019; Chen et al., 2021). Importantly, most studies agree that pathogenic changes in gut microbiota could contribute to HIV progression (Li et al., 2019; Vujkovic-Cvijin and Somsouk, 2019; Coleman et al., 2020; Chen et al., 2021).

A previous study investigated the sexual behaviors of 1691 MSM, in which 67.0% of participants had performed oroanal sex or rimming and 65.6% had used their partners’ saliva as an anal lubricant to aid fingering or penis dipping (Cornelisse et al., 2018). Furthermore, MSM with HIV-infected partners significantly favor saliva as an anal sex lubricant (Butler et al., 2009); however, lubricants may be an important factor contributing to MSM-HIV-associated gut dysbiosis (Vujkovic-Cvijin et al., 2020). Saliva remains an important vector of disease transmission during intercourse in MSM (Chow et al., 2016; Cornelisse et al., 2018; Mistry et al., 2022), even though oral sex has been identified to confer a lower risk of HIV transmission (Robinson and Evans, 1999; Scully and Porter, 2000; Campo et al., 2006). It follows that as commensals of HIV hosts with oroanal sex behavior, the salivary and gut microbiota are closely related.

Therefore, considering this background information, we hypothesize that unique features of sexual intercourse among MSM change salivary microbiota composition and we speculate that these compositional changes could profoundly influence the development of oral lesions in HIV-positive MSM. However, relevant studies on the salivary microbiota of HIV-positive MSM are currently limited. A recent study examining salivary bacterial diversity via 16S rRNA gene MiSeq sequencing revealed no differences in alpha diversity irrespective of HIV status, in which the controls were non-HIV-MSM, and antiretroviral therapy (ART) groups. ART decreased salivary diversity (Li et al., 2020). Our previous preliminary study focused solely upon MSM for analysis and examined the effect of HIV status by stratifying subjects into groups by disease state, as measured by the CD4 counts of the individuals. Interestingly, we came to a different conclusion than other published studies; we found that HIV infection resulted in greater salivary diversity, but AIDS did not. We also found that acute HIV infection resulted in a significant increase in salivary bacterial abundance (Guo et al., 2021a). We believe that it is precisely because of the oroanal sex behavior of MSM that a small number of seemingly contradictory studies have also initially examined the saliva microbial status of HIV-MSM, providing interesting perspectives for microbiome research.

16S rRNA gene sequencing is an approach most well-suited to exploring bacterial population taxonomy. Deeper analysis of the impacts of bacteria upon salivary functioning needs to be ascertained through other approaches, including metagenomics (Bienenstock et al., 2013). To date, we have retrieved only one metagenomics-based HIV periodontitis study that focused on oral bacterial changes rather than function and targeted the HIV population rather than HIV-MSM (Noguera-Julian et al., 2017). Therefore, it is necessary to deeply investigate the functional changes in oral microbiota in HIV-MSM through genetic analysis to reveal the subtle effects of oral microbiota on the occurrence and development of oral and related diseases under different immune states after HIV infection.

## 2 Materials and methods

### 2.1 Subject recruitment

The protocol was approved by the Institutional Review Board of Beijing Youan Hospital, Capital Medical University, and was registered at clinical trials.gov (ChiCTR2000030301). All participants signed written informed consent forms. Protocol-based inclusion criteria stipulated that only Chinese MSM greater than 18 years old who had anal or oral sex more than once in nearly 3 months could participate in the study. Exclusionary criteria included use of antibiotics, ART or immunomodulatory drugs within the past 3 months, history of any systemic disease, serious oral problems (not including non-cavitated caries, nonpurulent periodontal disease, or oral candidiasis), or possessing fewer than 20 teeth. Among the 106 MSM randomly selected from the Infectious Diseases Center of Beijing Youan Hospital, 55 MSM subjects were strictly screened according to the above criteria and subdivided into five groups based upon staging criteria for disease control and prevention of monitoring cases (Centers for Disease Control and Prevention (CDC), 2014). The five MSM groups consisted of an HIV-negative control group [n=11] and four HIV-positive subgroups [n=44]. The four HIV-positive subgroups were divided as follows: stage 0 possessed a positive result for HIV RNA but screened negatively (or indeterminately) for HIV antibody [n=11]; stage 1 (CD4 > 500) [n=10]; stage 2 (CD4: 200–500) [n=13]; stage 3 (either CD4 < 200 or opportunistic infection) [n=10].

### 2.2 Sample collection

Brief medical history inquiries, comprehensive oral examinations, and confirmation of MSM subject enrollment and HIV serostatus were conducted at the Department of Stomatology of Beijing Youan Hospital for all the enrolled subjects. The subjects were required to not eat, drink, or perform oral hygiene procedures 2 h before sampling, and spit 5 ml of saliva into sterile tubes that would be placed on ice in polystyrene plastic boxes and transferred to a -80°C refrigerator within 2 h.

### 2.3 16S rRNA gene sequencing and analysis

To reduce costs, we chose a two-stage microbial community experimental design (Tickle et al., 2013). Our previous article (Guo et al., 2021a) detailed our protocols for DNA extraction, PCR amplification, and Illumina MiSeq sequencing of 16S rRNA gene sequences and obtained salivary bacterial diversity data, which we used to perform MicroPITA (Microbiomes: Picking Interesting Taxonomic Abundance) analysis. We then selected 25 salivary samples (5 samples per group) for subsequent metagenomics studies in accordance with the most representative (i.e., reflecting overall species composition) and multiple selections (i.e., selecting samples by two or more methods including most dissimilar or maximum diversity, and most representative). Gene amplicon sequencing data have been published in the NCBI Sequence Read Archive database (SRP251412).

### 2.4 Metagenomic sequencing and analysis

Total genomic DNA was extracted from 25 salivary samples using the E.Z.N.A.® Soil DNA Kit (Omega Bio-tek, Norcross, GA, U.S.). DNA concentration and purity were determined using TBS-380 and NanoDrop2000 instruments. A sequencing library of 400 bp DNA fragments was constructed using a NEXTFLEX® Rapid DNA-Seq (Bioo Scientific, Austin, TX, USA), and sequencing was performed on Illumina NovaSeq PE150 platform (Illumina Inc., San Diego, CA, USA) at Majorbio Bio-Pharm Technology Co., Ltd. (Shanghai, China). Metagenomic sequencing data have been published within the NCBI Sequence Read Archive database (SRP327008). After performing sequence quality control and removing any reads from the human genome, we assembled metagenomic data using MEGAHIT (https://github.com/voutcn/megahit, version 1.1.2) and selected contigs with lengths ≥ 300 bp for further gene prediction and the Kyoto Encyclopedia of Genes and Genomes (KEGG) annotation analyses using Diamond (http://www.diamondsearch.org/index.php, version 0.8.35) alongside KEGG database (http://www.genome.jp/keeg/) with an e-value cutoff of 1e-5.

### 2.5 Statistical analysis

Statistical analyses were performed using SAS 9.4 (SAS Institute Inc., Cary, NC, USA). Nonparametric Kruskal-Wallis rank-sum tests and LDA linear discriminant analyses (LDA > 2) in LEfSe differential discriminant analyses between groups were used to assess differences in the functional abundances and effects of differentially represented KEGG functions, respectively. Among them, multi-group comparisons used an all-reverse-all strategy; that is, only differences observed in multiple groups may be properly considered as differential functions. Comparative metabolic pathway analysis between groups was applied using iPath 2.0 (http://pathways.embl.de).The correlation between CD4 counts, blood viral load (BVL), and salivary microbiota functions was assessed by calculating Spearman’s correlation coefficients. We considered *P* < 0.05 as our cutoff for statistical significance across all assays.

## 3 Results

### 3.1 MicroPITA analysis of 16S rRNA gene sequencing

After 16S rRNA gene sequencing, we obtained salivary microbiota diversity data and visualized lineages by performing cluster analyses (Guo et al., 2021a). Applying MicroPITA to these sequences, a total of 55 samples were identified, of which we selected the representative 25 for further metagenomic sequence analysis to allow differences between samples to be discerned.

### 3.2 KEGG functional composition of salivary bacterial microbiota

**Figure 1:**
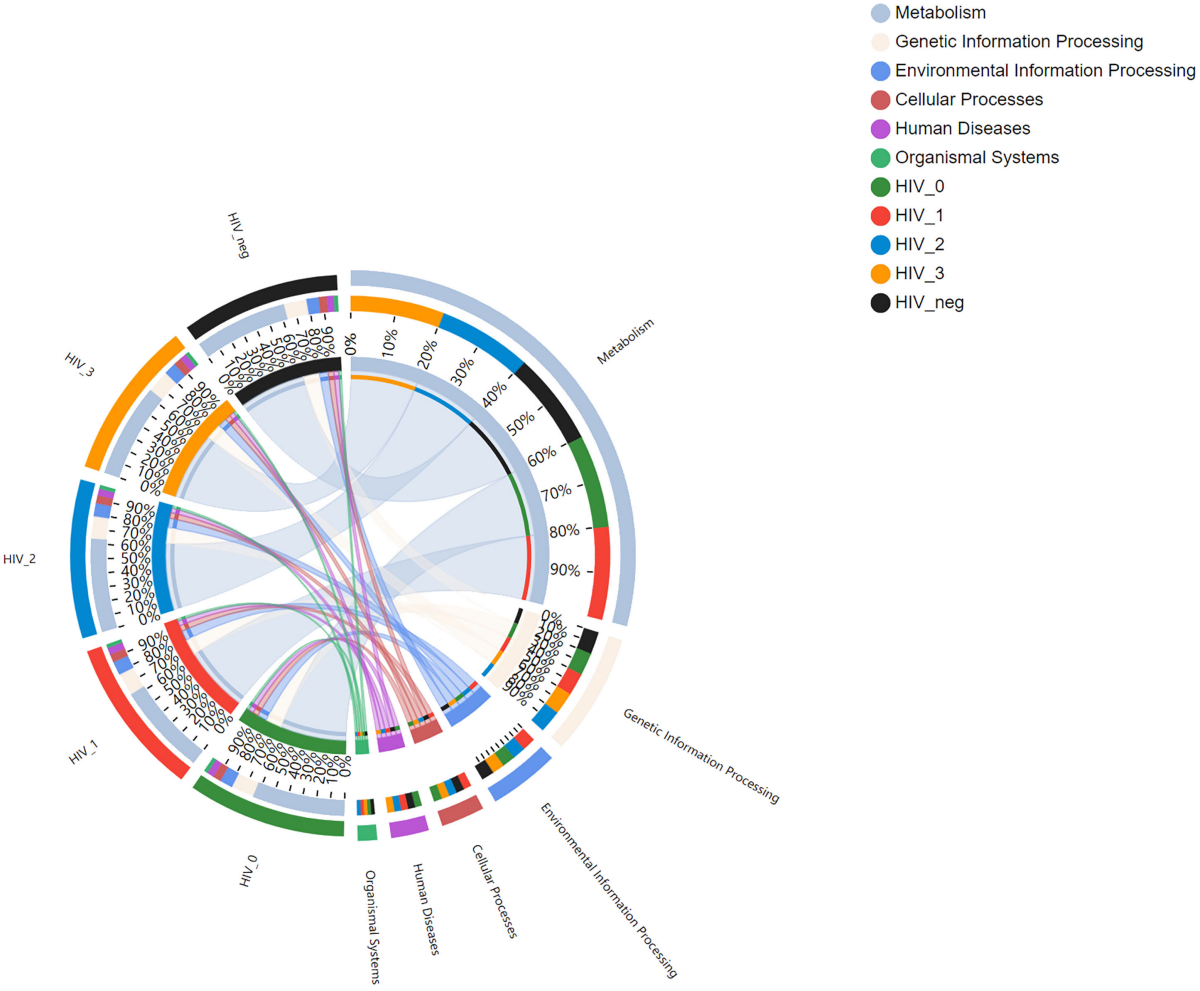
Functional Circos plot of salivary bacterial microbiota at Pathway Level 1. The left semicircle (smaller circle) represents the functional abundance composition of the five groups assayed: controls (black), stage 3 (orange), stage 2 (blue), stage 1 (red), and stage 0 (green). The right semicircle represents the distribution ratio of functions in different samples at the clustering level: metabolism (light blue), genetic information processing (pale pink), environmental information processing (blue), cellular processes (crimson), human diseases (purple) and organismal systems (green). From outside to inside, the left circles represent the functional compositions of different samples and the abundance ratios of different functions, respectively; as above, the right sides of the circles represent the distribution ratios of different samples with respect to their dominant functions. Inside the circles, the widths of the colored bars connecting samples (left) and functions (right) represent the relative abundances of the function within each sample and the distribution ratio of the sample in the corresponding function, respectively.

**Figure 2:**
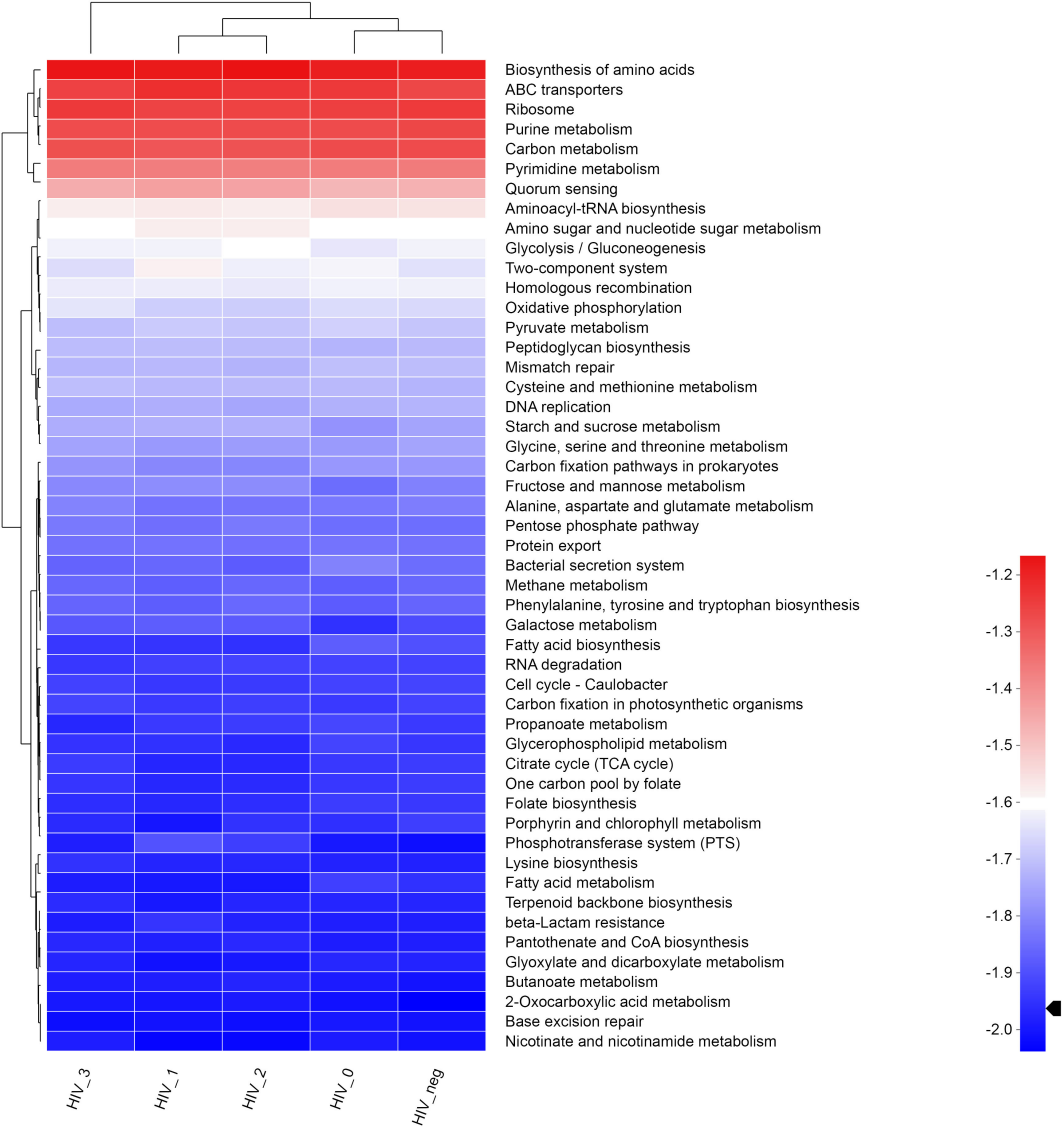
KEGG functional heatmap of salivary bacterial microbiota. Heatmap showing the top 50 most abundant functions, with shades of color representing functional abundance levers in the five groups at Pathway Level 3, and the functional clustering tree (left) and sample clustering tree (top) are also depicted along with a legend.

A total of 6, 45, and 325 functions at Pathway Level 1, 2, and 3, respectively, were identified according to KEGG PATHWAY analysis of higher-order functions (Kanehisa et al., 2006) and were annotated in MSM salivary samples using KOBAS 2.0 (KEGG Orthology Based Annotation System). Detailed information regarding the three pathway levels is shown in **Supplementary Table S1**. Subsequently, functional abundances were visualized using Circos plot (**Figure 1**), which showed that the most represented functions in each group, as well as the relative proportions of different functions, were almost identical among Pathway Level 1 functions. The main annotated functions are as follows: metabolism, genetic information processing, environmental information processing, cellular processes, human diseases, and organismal systems. Further, the top 50 most abundant functions at Pathway Level 3 were visualized using heatmaps (**Figure 2**). According to the sample which detailed information of three Pathway levels were shown in **Supplementary Table S1**. According to the sample clustering relationship analysis results plotted in the accompanying heatmap (**Figure 2**), we could tentatively identify functional changes between groups at Pathway Level 3, which were characterized by similar bacterial functions between stage 0-control and stage 1-2 groups, whereas the stage 3 group exhibited the greatest functional change.

### 3.3 KEGG functional differences among salivary bacterial microbiota

**Figure 3:**
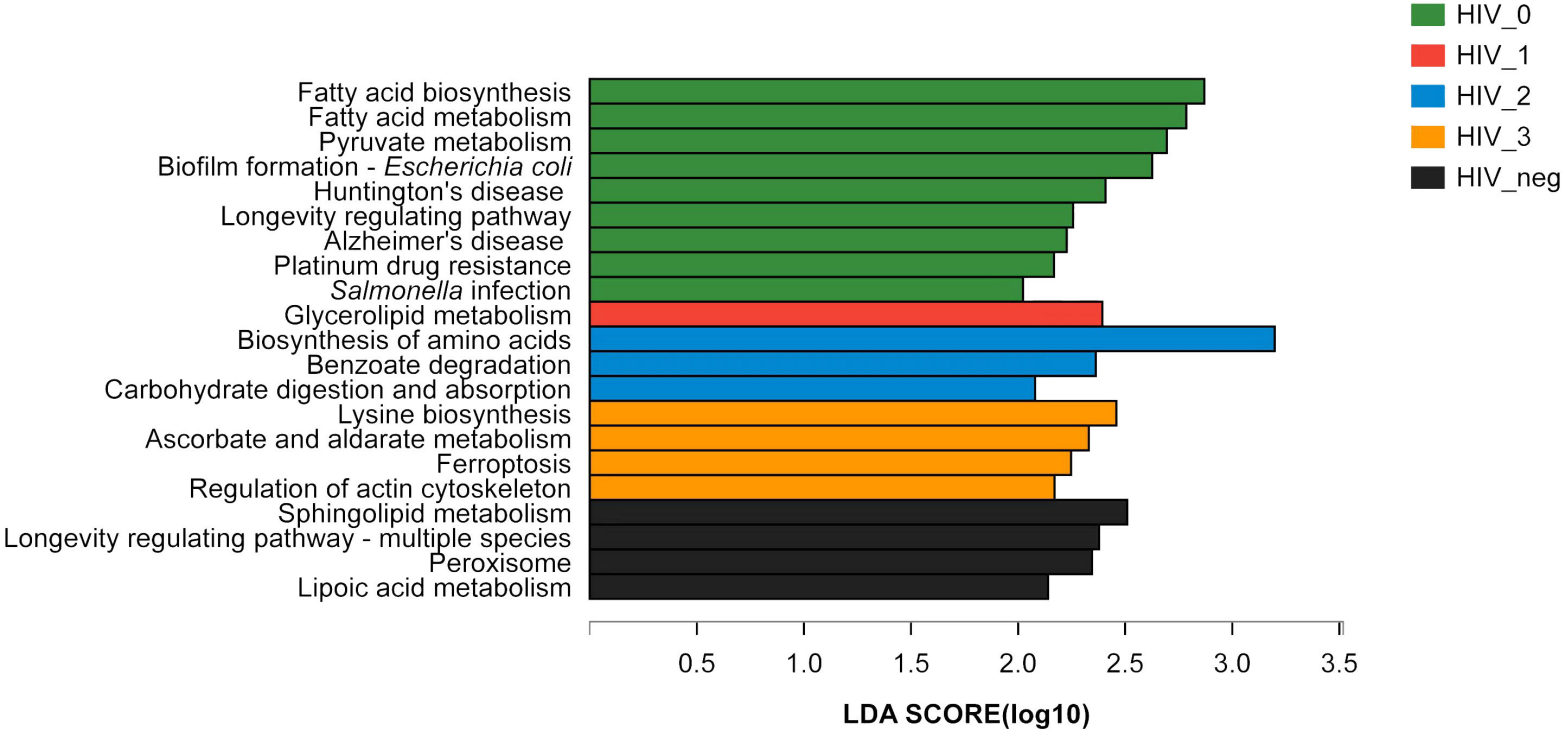
LEfSe bar graph of salivary bacterial functional differences between HIV-positive and HIV-negative control groups. An LEfSe bar graph of the functional differences between the salivary bacterial microbiota of HIV-positive and HIV-negative control groups (at KEGG Pathway Level 3) has been shown. Stage 0 (green), stage 1 (red), stage 2 (blue), stage 3 (orange), and controls (black).

The LEfSe Bar graph (**Figure 3**) depicts differentially annotated functions of salivary bacteria groups at KEGG Pathway Level 3, which is detailed in **Table 1**. We summarize the principal differences in the functional characteristics of the samples in the following description. (1) The stage 0 group had nine functions with abnormally increased abundance and was much higher than any of the other three HIV-positive groups assayed. Their abnormally increased functions included: fatty acid biosynthesis, fatty acid metabolism, pyruvate metabolism, biofilm formation, *Escherichia coli*, Huntington’s disease, longevity regulatory pathway, Alzheimer’s Disease, platinum resistance, and *Salmonella* infection, therefore mainly related to metabolism and human diseases. (2) Stage 1 had the fewest abundantly enriched functions, only one in fact, which was glyceride metabolism. Stages 2 and 3 had three (amino acid biosynthesis, benzoic acid degradation, carbohydrate digestion, and absorption) and four (lysine biosynthesis, ascorbic acid and aldonate metabolism, ferroptosis, and regulation of the actin cytoskeleton) annotated functions, respectively. (3) All four HIV-positive groups exhibited abnormal enrichments for different functions but shared common characteristic depletions in sphingolipid metabolism and longevity regulation pathways including multispecies, peroxisome, and lipoic acid metabolism.

**Table 1:** Differential KEGG functions in salivary bacterial microbiota between experimental groups.

KEGG level	Lever 3	Lever 2	Lever 1	LDA value	*P* value
HIV_0	Fatty acid biosynthesis	Lipid metabolism	Metabolism	2.87025	0.00768
	Fatty acid metabolism	Global and overview maps	Metabolism	2.78537	0.01056
	Pyruvate metabolism	Carbohydrate metabolism	Metabolism	2.69462	0.01802
	Biofilm formation - *Escherichia coli*	Cellular community - prokaryotes	Cellular Processes	2.62640	0.03466
	Huntington's disease	Neurodegenerative diseases	Human Diseases	2.40849	0.00905
	Longevity regulating pathway	Aging	Organismal Systems	2.25741	0.01097
	Alzheimer's disease	Neurodegenerative diseases	Human Diseases	2.22721	0.03510
	Platinum drug resistance	Drug resistance: Antineoplastic	Human Diseases	2.16824	0.03959
	*Salmonella* infection	Infectious diseases: Bacterial	Human Diseases	2.02329	0.02492
HIV_1	Glycerolipid metabolism	Lipid metabolism	Metabolism	2.39419	0.03015
HIV_2	Biosynthesis of amino acids	Global and overview maps	Metabolism	3.19791	0.04328
	Benzoate degradation	Xenobiotics biodegradation and metabolism	Metabolism	2.36292	0.02798
	Carbohydrate digestion and absorption	Digestive system	Organismal Systems	2.07978	0.00464
HIV_3	Lysine biosynthesis	Amino acid metabolism	Metabolism	2.45905	0.01247
	Ascorbate and aldarate metabolism	Carbohydrate metabolism	Metabolism	2.33079	0.01969
	Ferroptosis	Cell growth and death	Cellular Processes	2.24746	0.04314
	Regulation of actin cytoskeleton	Cell motility	Cellular Processes	2.17035	0.00775
HIV_neg	Sphingolipid metabolism	Lipid metabolism	Metabolism	2.50975	0.02806
	Longevity regulating pathway - multiple species	Aging	Organismal Systems	2.37896	0.00697
	Peroxisome	Transport and catabolism	Cellular Processes	2.34590	0.04032
	Lipoic acid metabolism	Metabolism of cofactors and vitamins	Metabolism	2.14090	0.00579

### 3.4 Relationship between CD4 count, BVL, and function of salivary bacteria in HIV-positive subjects

**Figure 4:**
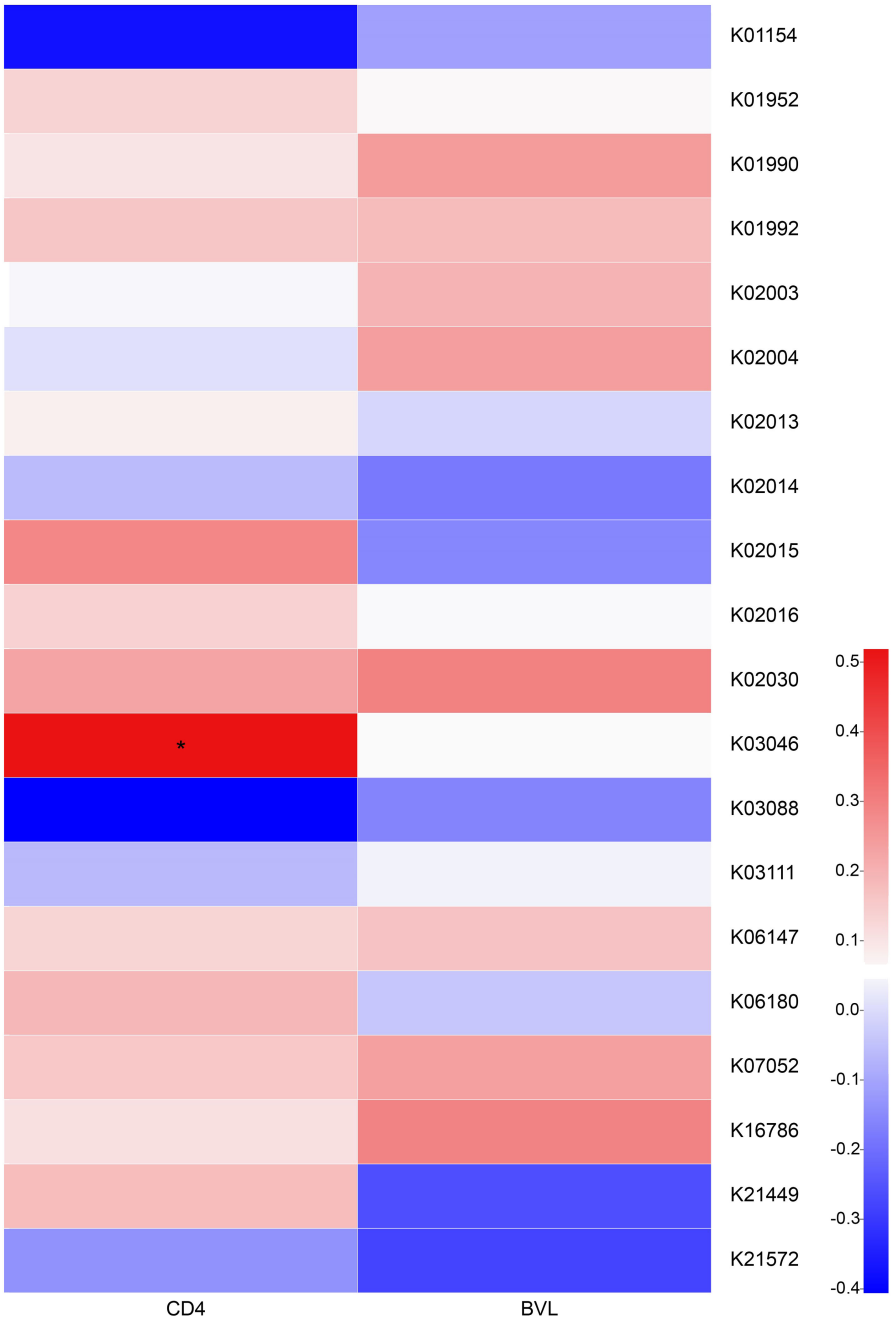
Correlation between the top 20 abundant KOs of HIV-positive salivary bacteria and BVL, CD4 counts. A correlation heatmap of the relationship between the top 20 abundant KOs of salivary bacteria and BVL, or CD4 counts, in the HIV-positive groups is shown. The colors represent different calculated r values (Spearman correlation coefficient). Red and blue colors indicate positive and negative correlations, respectively. *0.01 < P ≤ 0.05.

We next prepared a correlation heatmap (**Figure 4**) to explore the effects of CD4 counts and BVL upon the functions of HIV-positive salivary microbiota. We found no significant correlation between KEGG Orthology (KO) and BVL in the top 20 genes related to salivary function; however, K03046 was positively correlated with increasing CD4 counts (*r* = 0.5188, *P* = 0.01909). K03046 (*rpoC* gene) is annotated as DNA-directed RNA polymerase subunit beta.

### 3.5 Metabolic pathway differences among salivary bacterial microbiota in MSM

**Figure 5:**
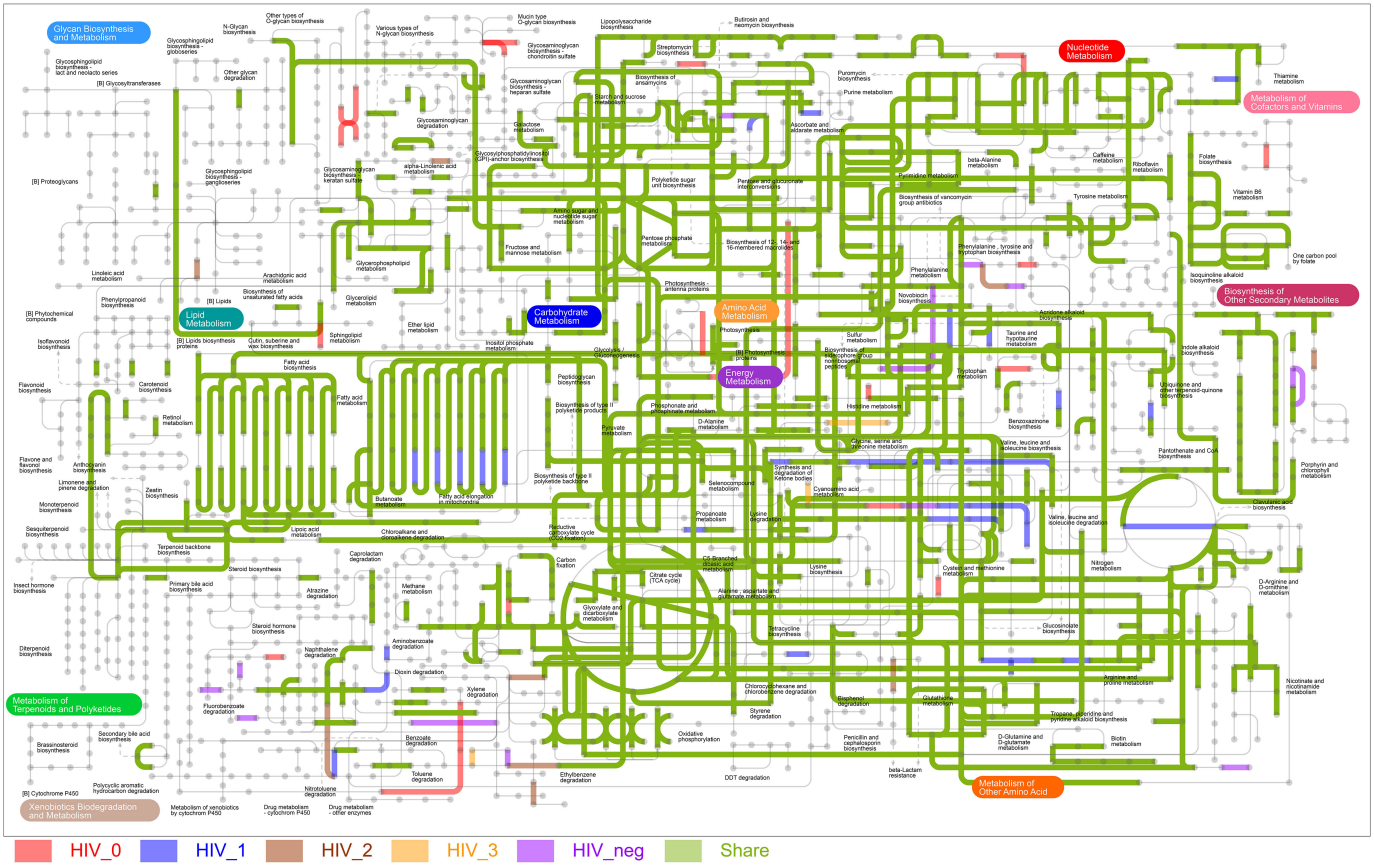
The ipath metabolic pathway map of MSM salivary microbiota. Line segments with colors represent different enzymatic or metabolic pathways. Shared and unique enzymatic and metabolic pathways are colored as follows: shared (green); HIV-negative control group unique (purple), stage 0 group unique (red), stage 1 group unique (blue), stage 2 group unique (brown), and stage 3 group unique (orange).

The metabolic pathway map of MSM salivary microbiota (**Figure 5**) visualizes the annotated enzymes and metabolic pathways noted for each group. In general, most of the pathways we identified were common to both the HIV-positive and -negative control groups, but some KEGG metabolic pathways did uniquely appear in certain groups. The stage 0 group was most abundant in unique pathways, xylene degradation and [B] photosynthesis proteins related pathways most prominent. In stage 1, enrichment in fatty acid elongation in the mitochondria was a particularly striking feature. The number of characteristic pathways in stage 2 and 3 was considerably lower.

## 4 Discussion

It is generally believed that HIV infection is often comorbid with a variety of oral diseases, which likely occur due to perturbations to the close association between commensal oral microbiota diversity, functional composition and the host (Kistler et al., 2015; Noguera-Julian et al., 2017; Weinberg et al., 2020; Li et al., 2021; Guo et al., 2021b). However, a comprehensive exploration of these relationships has yet to be completed. To further expand our inquiries and obtain a fuller panorama of salivary microecology across HIV stages, here we have explored the functions of salivary microbiota across multiple stages of disease progression and performed comparisons between stages to glean metagenomic insights into the specific perturbing effects of HIV status on the salivary microbial communities of MSM.

Our previous study on salivary microbial diversity in HIV infection described and analyzed the taxonomy, composition, and characterization of salivary microbiota changes at different stages of HIV-MSM infection, finding that HIV-positive saliva had significantly greater diversity (excluding AIDS stage) and abnormally proliferating bacteria increased in numbers dramatically during the acute HIV infection (Guo et al., 2021a). To extend our findings, here we have applied metagenomics to analyze the genetic functional levels of 25 representative saliva samples, screened by MicroPITA, to reveal latent “information” by analyzing changes in salivary microecologies brought about by MSM and HIV statuses, as these changes may induce changes that drive oral diseases. Our study found that metabolism, genetic and environmental information processing, and cellular processes were the core salivary bacterial functions at KEGG Pathway Level 1, with HIV-positive and -negative control MSM showing mostly the same functional composition at this level. Further, we found that the top 5 most abundant functions at Pathway Level 3 were biosynthesis of amino acids, ABC transporters, Ribosome, Purine metabolism and Carbon metabolism. Moreover, salivary function enrichment at different infection stages revealed interesting convergent and divergent characteristics among assayed groups. Cluster analysis of the accompanying heatmap showed that salivary bacterial microbiota exhibit a slow, functional transition as the post-HIV exposure time lengthens. That is, that MSM with acute infections (stage 0) exhibited parameters close to the parameters we observed for negative controls. However, as host immunosuppression becomes progressively aggravated, functional differences between the stages begin to emerge. Salivary functions of the two groups in the asymptomatic stage (stages 1 and 2) continued to be similar, but for the AIDS stage (stage 3), apparent functional differences again markedly increased compared to the other four groups and was the most significantly different from the negative controls. Therefore, we hypothesize that the functions of salivary bacterial microbiota in untreated HIV-MSM slowly and progressively alter as the disease progresses and eventually manifests markedly dysfunctional imbalances at advanced stages.

The KEGG functions of salivary bacterial microbiota varied along the course of the disease, and each stage had unique functional characteristics. First, abnormal hyperfunction was most prominent in the acute infection stage, mainly involving energy metabolism, infection damage, and drug resistance. Enhanced metabolism of lipids and carbohydrates could be associated with energy expenditure due to the energetic costs of HIV invasion. Abnormal hyperactivity of biofilm formation in *Escherichia coli* and *Salmonella* infection, Huntington’s disease, and Alzheimer’s Disease has targeted vulnerable target organs in the early stage of HIV infection, such as the nervous system and the intestine, which is also consistent with the clinical manifestations (Vanhems et al., 1999; Valcour et al., 2012). The early tendency of nerve damage occurring deserves attention, and the abnormally high enhancement of intestinal function may be related to particular intercourse endemic to MSM. Platinum drug resistance indicates that antiviral and antitumor chemoresistance has emerged earlier, and early depletion of longevity-regulating pathways indicates that early intervention with ART is necessary. In short, the functional expression of salivary microbiota during acute infection is of great significance for understanding the early control of the disease. Secondly, HIV-MSM in the mildly immunosuppressed stage had the fewest functional abnormalities, and their overall status was fairly stable, except for their augmented level of glycerolipid metabolism we annotated. Thirdly, during the stage of moderate immunosuppression, abnormally hyperactivated functions began their increases and still manifest as changes in metabolism and digestion functional enrichments, such as in biosynthesis of amino acids, benzoate degradation, and carbohydrate digestion and absorption pathways. Fourth, the abnormal functions at the severely immunosuppressed stage increased further and was again mainly reflected in changes to the levels of metabolism and cellular processes. Hyperfunction of lysine biosynthesis can prevent herpes labialis or oral ulcers and accelerate tissue repair (Spallotta et al., 2013; Mailoo and Rampes, 2017). Induction of ferroptosis may attenuate carcinogenesis (Zhu et al., 2019), whereas chronic periodontitis and human papillomavirus infection are associated with ascorbate and aldarate metabolism and regulation of the actin cytoskeleton (Chowdhry et al., 2019). The above information indicates that with the massive depletion of CD4^+^T lymphocytes, oral microbiota may stabilize the balance of the oral ecological environment by attempting to compensate for the host’s deficient functions. Finally, vigorous energy metabolism, infection, and changes in cellular immunity are the same as shown in the metabolic research analysis of HIV-infected individuals (Sitole et al., 2013), and are consistent with clinical characteristics of high consumption and weight loss prevalent in clinical HIV-positive individuals (Sharpstone et al., 1996), especially at the acute stage. However, HIV-MSM saliva showed functional attenuation as well in the following four aspects: sphingolipid metabolism involved in cell growth regulation (Spiegel and Merrill, 1996), longevity regulating pathway - multiple species, cell-protecting peroxisome, and lipoic acid metabolism that inhibits HIV replication and improves mitotic function of T lymphocytes (Baur et al., 1991; Jariwalla et al., 2008). Therefore, HIV may be closely related to the mechanism of apoptosis induction and utilizing the host biosynthetic machinery for survival, which deserves in-depth study and discussion.

We observed that the *rpoC* RNA polymerase gene that drives bacterial transcription (Lee et al., 2013) was strongly positively correlated with increasing CD4 counts. This may mean that the expression of certain bacterial genes gradually weakened with the decline in host immune status in HIV-MSM, which in turn impairs the balance of the oral microecological environment. Among the KEGG metabolic pathways of the five groups, salivary microbiota at stage 0 showed a relatively high abundance of unique pathways, which were identified in xylene degradation and [B] photosynthesis proteins, indicating microbiota during the acute infection stage increased their energy supply and degradation of toxic substances, while enrichment of fatty acid elongation in mitochondria during mild immunosuppression provided pathways for the enhanced production of essential fatty acids that the body cannot synthesize. The results once again demonstrate that HIV infection might promote abnormal host energy metabolism and pathogen virulence.

Our study further obtained metagenomic profiles of salivary microbiota from twenty different HIV-positive and five HIV-negative MSM, based on 16S rRNA data. However, the study has a few limitations. Our small sample size limited our ability to representatively sample the overall HIV-positive MSM population of China. We also lacked important covariate data, including information on oral hygiene habits, smoking status, and sexual intercourse habits. Cross-sectional analyses limited our analytical perspective because of the ethics of necessarily providing immediate standard treatment for HIV, which prevented us from conducting cohort observational studies of different infectious stages in untreated HIV-MSM.

## 5 Conclusion

In summary, the most abundant functions annotated in HIV-positive salivary microbiota in untreated HIV-MSM appeared largely the same as those in healthy controls, mainly regulated metabolism and genetic or environmental information processing. With disease progression, salivary functions exhibited slow and progressive changes and showed significant dysfunction on reaching the AIDS stage, which was functionally distinct from other stages we assayed. The abnormally proliferating KEGG functions annotated to HIV-MSM salivary microbiota were characterized by high-energy metabolism and restricted cellular regulation. Infection and drug resistance of acute stage and immune cell destruction of AIDS stage were abnormally increased, predicting an increased risk for such individuals to develop systemic and oral diseases. Preliminary discussion on metabolic pathways and characteristic enzymes also supported our theory that salivary microbiota participates in and maintains homeostasis of the oral microenvironment, and that HIV infection might promote abnormal energy metabolism and exacerbate pathogen virulence.

## Data availability statement

The datasets presented in this study can be found in online repositories. The names of the repository/repositories and accession number(s) can be found below: https://www.ncbi.nlm.nih.gov/, SRP251412 https://www.ncbi.nlm.nih.gov/, SRP327008.

## Ethics statement

The studies involving humans were approved by The Institutional Review Board of Beijing Youan Hospital, Capital Medical University. The studies were conducted in accordance with the local legislation and institutional requirements. The participants provided their written informed consent to participate in this study. Written informed consent was obtained from the individual(s) for the publication of any potentially identifiable images or data included in this article.

## Author contributions

YG: Writing – original draft, Writing – review & editing, Conceptualization, Data curation, Investigation, Methodology, Software. WW: Writing – review & editing, Data curation, Project administration and Resources. YY: Writing – review & editing, Data curation. XS: Data curation, Writing – review & editing. BZ: Data curation, Writing – review & editing. YW: Data curation, Writing – review & editing. JC: Data curation, Writing – review & editing. SW: Data curation, Writing – review & editing. XW: Data curation, Writing – review & editing, Conceptualization. YL: Data curation, Writing – review & editing, Validation. SC: Formal analysis, Methodology, Writing – review & editing, Conceptualization. RW: Writing – review & editing. WD: Writing – review & editing, Investigation, Data curation. WX: Investigation, Writing – review & editing. FW: Formal analysis, Writing – review & editing. JD: Project administration, Writing – review & editing. HD: Investigation, Writing – review & editing. SG: Project administration, Writing – review & editing. FZ: Writing – review & editing, Writing – original draft. ZS: Writing – review & editing, Writing – original draft, Conceptualization. XH: Writing – original draft, Writing – review & editing.
